# OsNHX5-mediated pH homeostasis is required for post-Golgi trafficking of seed storage proteins in rice endosperm cells

**DOI:** 10.1186/s12870-019-1911-y

**Published:** 2019-07-05

**Authors:** Jianping Zhu, Yulong Ren, Yunlong Wang, Feng Liu, Xuan Teng, Yuanyan Zhang, Erchao Duan, Mingming Wu, Mingsheng Zhong, Yuanyuan Hao, Xiaopin Zhu, Jie Lei, Yongfei Wang, Yanfang Yu, Tian Pan, Yiqun Bao, Yihua Wang, Jianmin Wan

**Affiliations:** 1 State Key Laboratory of Crop Genetics and Germplasm Enhancement Jiangsu Plant Gene Engineering Research Center, Nanjing Agricultural University, Nanjing, 210095 China; 2 National Key Facility for Crop Resources and Genetic Improvement Institute of Crop Science, Chinese Academy of Agricultural Sciences, Beijing, 100081 People’s Republic of China; 3 College of Life Sciences, Nanjing Agricultural University, Nanjing, 210095 People’s Republic of China

**Keywords:** Glutelin, DV, PBII, OsNHX5, Rice

## Abstract

**Background:**

As the major storage protein in rice seeds, glutelins are synthesized at the endoplasmic reticulum (ER) as proglutelins and transported to protein storage vacuoles (PSVs) called PBIIs (Protein body IIs), where they are cleaved into mature forms by the vacuolar processing enzymes. However, the molecular mechanisms underlying glutelin trafficking are largely unknown.

**Results:**

In this study, we report a rice mutant, named *glutelin precursor accumulation6* (*gpa6*), which abnormally accumulates massive proglutelins. Cytological analyses revealed that in *gpa6* endosperm cells, proglutelins were mis-sorted, leading to the presence of dense vesicles (DVs) and the formation paramural bodies (PMBs) at the apoplast, consequently, smaller PBII were observed. Mutated gene in *gpa6* was found to encode a Na^+^/H^+^ antiporter, *OsNHX5*. *OsNHX5* is expressed in all tissues analyzed, and its expression level is much higher than its closest paralog *OsNHX6*. The OsNHX5 protein colocalizes to the Golgi, the *trans*-Golgi network (TGN) and the pre-vacuolar compartment (PVC) in tobacco leaf epidermal cells. In vivo pH measurements indicated that the lumens of Golgi, TGN and PVC became more acidic in *gpa6*.

**Conclusions:**

Our results demonstrated an important role of OsNHX5 in regulating endomembrane luminal pH, which is essential for seed storage protein trafficking in rice.

**Electronic supplementary material:**

The online version of this article (10.1186/s12870-019-1911-y) contains supplementary material, which is available to authorized users.

## Background

Rice seeds accumulate large amount of storage proteins, including glutelin, prolamin, and α-globulin, which supply nutrients for seed germination and seedling growth. Up to 80% of the total seed storage proteins are made up of glutelins which are important protein sources for human consumption because of their easy digestibility. Glutelins are synthesized as 57 kD precursors on the rough endoplasmic reticulum (RER) and transported to PBIIs by DV-mediated post-Golgi transport pathway or ER-derived precursor-accumulating compartments [1–6].

Rice 57H mutants are characterized by over-accumulation of 57 kD glutelin precursors in seeds, which are excellent genetic resources to dissect the glutelin vacuolar transport pathway. Up till now, nine 57H mutants have been reported in rice, including *gpa1*/*glup4*, *gpa2*/*glup6, gpa3, gpa4*/*glup2, W379*/*glup3*, *esp2*, *glup1*/*esp5*, *Glup5*, *and glup7* [5–20]. Among which four cloned genes have been shown to regulate the proglutelin trafficking events. *GPA4* encodes GOLGI TRANSPORT 1B which regulates protein export from the ER [17, 20]. GPA3, a plant-specific Kelch-repeat domain containing protein, acts as a scaffold to recruit guanine-nucleotide exchange factor (GEF) GPA2/OsVPS9A, which in turn activates a small GTPase GPA1/OsRab5a [5, 6, 16, 18, 19]. GPA3, GPA2, and GPA1 proteins form a functional complex on the DVs to regulate the vacuolar trafficking of proglutelins [6]. In addition, *ESP2* encodes protein disulfide isomerase-like1–1 (PDIL1–1) which regulates the disulfide bond formation in ER [3]. *W379* encodes a vacuolar processing enzyme which processes proglutelins into acidic and basic subunits in the PBII [8, 15]. Despite these advances, molecular mechanisms underlying glutelin trafficking are still elusive.

Na^+^/H^+^ antiporters (NHX antiporters) are H^+^-coupled cotransporters that transfer Na^+^ or K^+^ across membrane in exchange for H^+^ [21]. In plants, NHX antiporters are essential for cellular pH and ion homeostasis. They play important roles in various cellular processes, such as Na^+^, K^+^ movement, pH homeostasis, vesicular trafficking and protein targeting, stress response, plant growth and development [22–27]. Based on the subcellular localization, Arabidopsis NHX antiporters are classified into three subgroups which localized to the vacuoles (AtNHX1–4) [28–30], plasma membrane (AtNHX7/8) [24, 30, 31], and endosomal compartments (AtNHX5/6) [32], respectively. Particularly, AtNHX5 and AtNHX6 are localized at the Golgi, TGN and required for cell expansion, response of salt stress as well as vesicular trafficking [32]. These two proteins are also localized at the PVC and important for maintaining endomembrane luminal pH and receptor-mediated protein trafficking to the vacuole [33]. In addition, AtNHX5 and AtNHX6 are require for seed storage protein processing [34]. In rice, overexpression of *OsNHX1* enhanced tolerance to salt stress [35]. However, the function of endosomal NHX proteins in rice remain largely unknown.

In this study, we report the functional characterization of a rice *gpa6* mutant that accumulated a large amount of proglutelins in the mutant endosperm cells, and demonstrate that *GPA6* encodes a Golgi-, TGN- and PVC-localized Na^+^/H^+^ antiporter OsNHX5 which is essential for endomembrane luminal pH homeostasis and proglutelin vacuolar trafficking.

## Results

### *gpa6* seeds accumulate proglutelins and develop abnormal endosperm

**Fig. 1:**
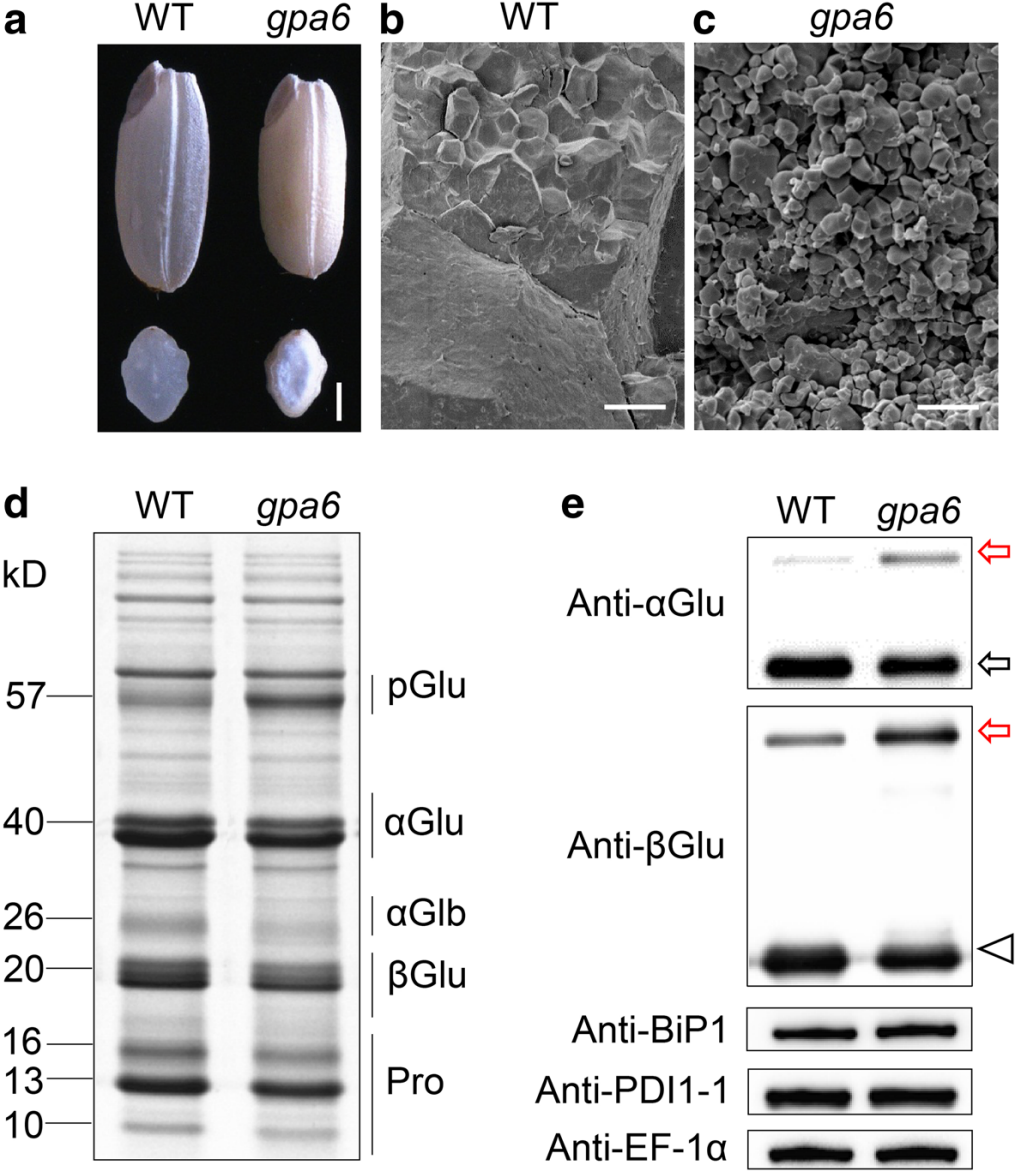
Characterization of the *gpa6* Mutant. **a** Transverse sections of representative wild-type (*Indica* cultivar N22) and *gpa6* mutant dry seeds. Bars = 1 mm. **b** and **c** Scanning electron microscopy images of transverse sections of wild-type (**b**) and *gpa6* mutant (**c**) seeds. Bars = 10 μm. **d** SDS-PAGE profiles of total seed storage proteins of the wild type and *gpa6* mutant. pGlu, 57-kD proglutelins; αGlu, 40-kD glutelin acidic subunits; αGlb, 26-kD α-globulin; βGlu, 20-kD glutelin basic subunits; Pro, prolamins. **e** Immunoblot analysis of glutelins and the molecular chaperones BiP1 and PDI1–1. Arrowheads represent glutelin basic subunits. Arrows indicate the 57-kD proglutelins (red) and the glutelin acidic subunits (black). EF-1α was used as a loading control

A 57H mutant named *gpa6* was isolated during our continuous effort to dissect the glutelin trafficking pathway in rice. Unlike the transparent endosperm of wild type, *gpa6* mutant endosperm appeared floury (Fig. 1a). Scanning electron microscopy (SEM) analysis revealed that *gpa6* endosperm comprised round and loosely packaged compound starch granules instead of the tightly packaged, crystal-like structures observed in the wild type (Fig. 1b and c). Meanwhile, the 1000-grain weight was significantly decreased, and the amylose content was reduced approximately 20% and the lipid content was increased 81% in the *gpa6* mutant. However, the total protein content in the endosperm was not changed (Additional file 8: Table S1). Compared with the wild type, *gpa6* accumulated much higher level of unprocessed 57 kD proglutelins, accompanied by concomitant reduction of both 40 kD acidic and 20 kD basic subunits of the mature glutelins (Fig. 1d). The appearance of higher amount of proglutelins was further confirmed by immunoblotting using antibodies against 40-kD glutelin acidic subunits and 20-kD glutelin basic subunits (Fig. 1e). Time course studies revealed that the abnormal accumulation of proglutelins in *gpa6* seeds occurred from ~ 12-DAF onwards (Additional file 1: Figure S1). In addition, the expression of representative genes coding for storage proteins, and the protein level of ER lumen BINDING PROTEIN1 (BiP1) and PDI1–1 showed almost no differences between *gpa6* and wild-type seeds (Fig. 1e, Additional file 2: Figure S2), indicating that the accumulation of 57 KD proglutelins was not due to an increasement of storage protein coding gene expression and probably protein synthesis was normal as no ER stress was detected. Collectively these data suggested that in the *gpa6* mutant might be defective in the trafficking of proglutelins.

### The *gpa6* mutant is defective in post-Golgi trafficking of storage proteins in developing endosperm

**Fig. 2:**
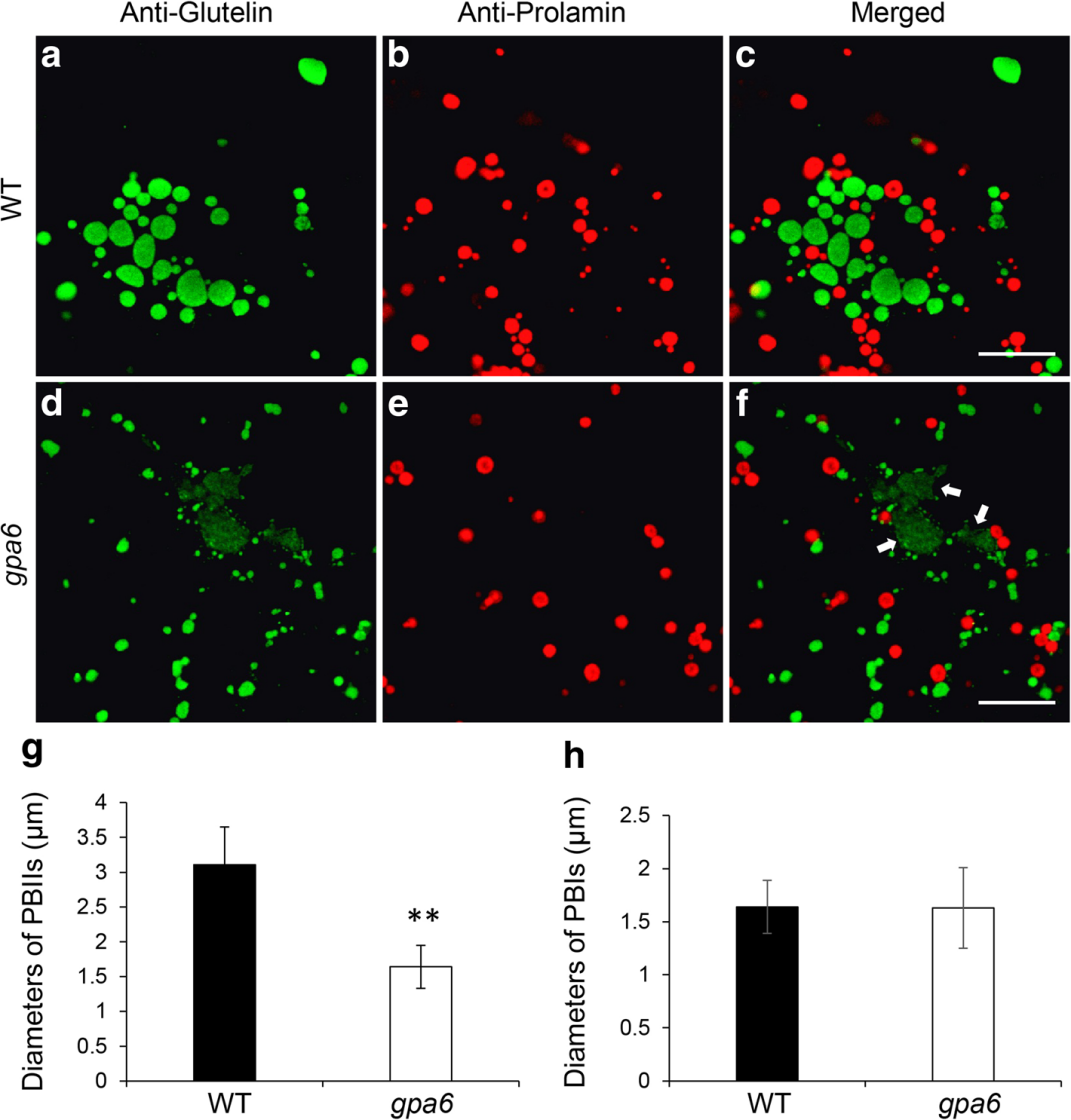
Immunofluorescence microscopy of protein bodies in the subaleurone cells of the wild type and *gpa6* mutant. **a** to **f** Immunofluorescence microscopy images of storage proteins in wild-type (**a**-**c**) and *gpa6* (**d**-**f**) 12 DAF seeds. **a**, **d** Secondary antibodies conjugated with Alexa fluor 488 (green) were used to trace the antigens recognized by the anti-glutelin antibodies. (**b**, **e**) Secondary antibodies conjugated with Alexa fluor 555 (red) were used to trace the antigens recognized by the anti-prolamin antibodies. (**c**, **f**) Merged images. White arrows in (**f**) indicate the PMB structures. Bars = 10 μm (**a**-**f**). (**g**) and (**h**) Statistical analysis of the diameters of PBIIs (g) and PBIs (h). Values are means ± SD. **P < 0.01 (*n* > 350, Student’s *t* test)

To gain an overview of glutelin deposition, semi-thin sections (1 μm) of 12-DAF wild-type and *gpa6* mutant endosperm were prepared and subjected to the immunofluoresence staining with specific antibodies against prolamins and glutelin acidic subunits (Fig. 2). In *gpa6* mutant, the PBIIs (containing glutelins) were reduced to 53% of the wild type (Fig. 2a-f, g), while the PBI sizes (containing prolamins) were comparable (Fig. 2a-f, h). Large glutelin-containing paramural bodies (PMBs) were readily observable (Fig. 2f). In addition, α-globulins were also transported incorrectly to the PMBs rather than PBIIs in *gpa6* (Additional file 3: Figure S3). Consistent with the proglutelin trafficking defects, Pectins labeled with JIM7 were seen to accumulate inside the PMBs in *gpa6* rather than display an even distribution along the wild type cell wall (Additional file 4: Figure S4) [36].

**Fig. 3:**
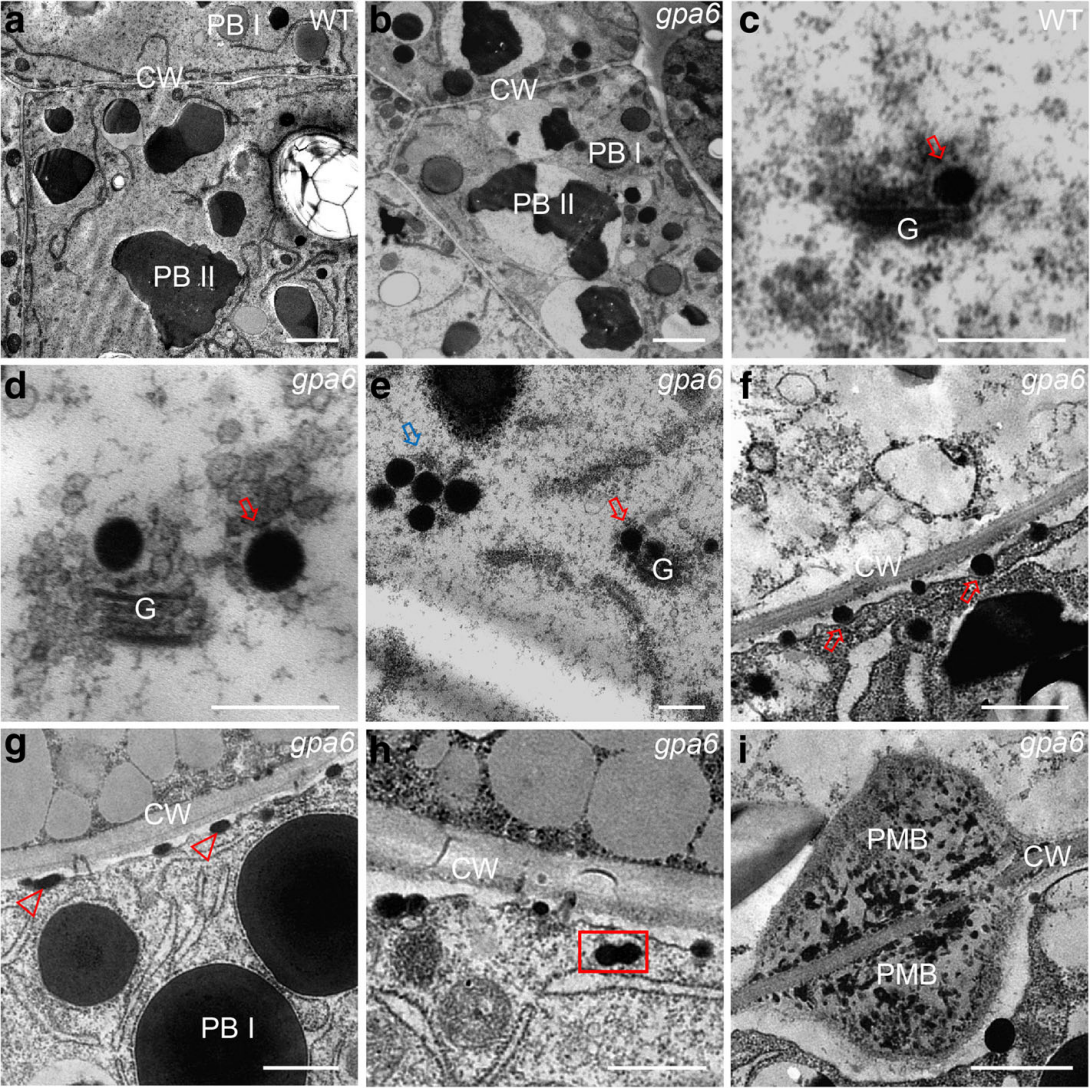
Ultrastructure of subaleurone cells of developing endosperm of the wild type and *gpa6* mutant. **a** and **b** Two types of protein bodies were observed in wild-type (**a**) and *gpa6* mutant (**b**) endosperm. Bars = 2 μm. CW, cell wall. **c** Wild type. Bars = 400 nm. **d** DVs bud off from the Golgi in the *gpa6* mutant. Red Arrow indicates enlarged DVs. Bars = 400 nm. **e** Large clusters of DVs (blue arrow) in the *gpa6* mutant. Bars = 400 nm. **f** and **g** Electron micrographs showing that DVs can fuse with the PM (**f**) and expel their contents into the apoplast forming oval-shaped structures (Arrowheads) (**g**) in the *gpa6* mutant. Bars = 1 μm. **h** Two DVs are fused with each other (rectangular box) in the *gpa6* mutant. Bars = 1 μm. (i) The PMB structures in the *gpa6* mutant. Bars = 1 μm

**Fig. 4:**
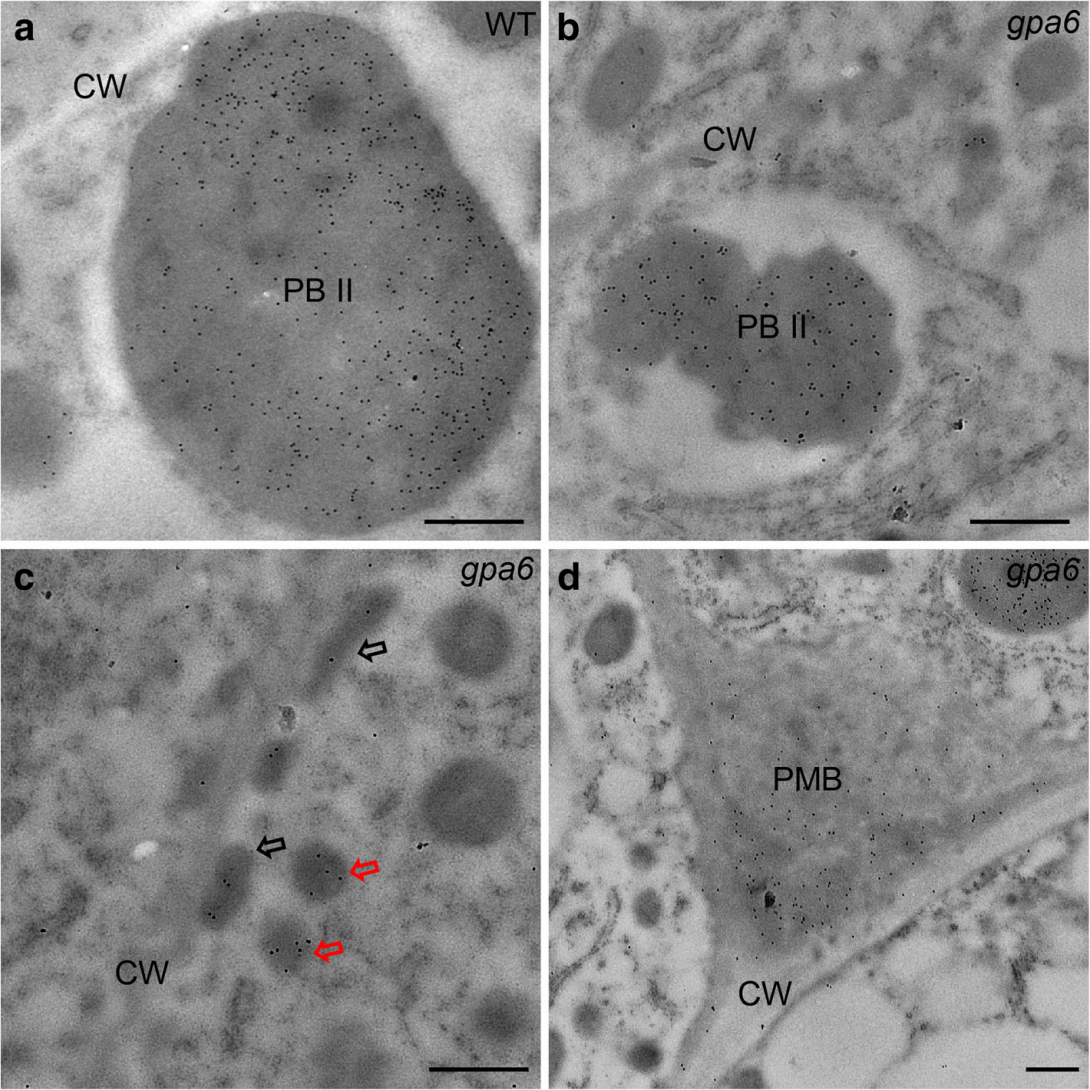
Immunoelectron microscopy localization of glutelins in rice endosperm cells. **a** Glutelins were accumulated in PBIIs in the wild-type endosperm cells. CW, cell walls. Bars = 500 nm. **b** Size-reduced PBII containing glutelins. Bars = 500 nm. **c** Glutelins in DVs (red arrows) and oval-shaped structures (black arrows). Bars = 500 nm. **d** Glutelins in the PMBs. Bars = 500 nm. 10-nm gold particle conjugated secondary antibodies were used in (a–d)

Next, subcellular observation by transmission electron microscopy (TEM) was performed using ultra-thin sections of 12-DAF developing endosperm. In wild-type endosperm cells, there are irregularly shaped, fully filled PBIIs and round spherical PBIs (Fig. 3a), however, in the *gpa6* mutant, PBIIs were only partially filled with the storage proteins (Fig. 3b), which was accompanied by the presence of PMBs (formed by the clustered DVs) and secreted oval-shaped structures along the cell wall (Fig. 3g and i). To determine structural alterations in the endomembrane system that could account for the missorting of storage protein precursors to the apoplast, we analyzed 12-DAF developing endosperm by immunogold labeling. Indeed, in the *gpa6* mutant, only part of glutelins was correctly transported to the PBIIs (Fig. 4a and b), meanwhile, mis-sorted DVs and PMBs were found to contain glutelins (Fig. 4c and d). These results demonstrated that proglutelin were mis-sorted, and delivered to the apoplast.

Proglutelins are delivered to the PBII via DV-mediated trafficking pathway [5, 6], therefore, DV morphology are carefully examined. Statistical analysis showed that the average diameter of DVs near the Golgi apparatus, and presumably just budding from the Golgi apparatus in *gpa6* is 193 nm (*n* = 75, Fig. 3d), which is larger than that of wild type (average 154 nm, *n* = 56, Fig. 3c), and these DVs tends to cluster in the cytosol (Fig. 3e). In addition, numerous DVs at an average diameter of 198 nm were found just fused with the plasma membrane, and kept spherical shapes (Fig. 3f), gradually, the secreted storage proteins became oval-shaped (Fig. 3g). Occasionally, fusion between two DVs was detected (Fig. 3h), this might account for the slight size increasement of DVs along the plasma membrane (198 nm) versus newly budded ones (193 nm). In short, DVs in *gpa6* mutant are enlarged.

Altogether, these results demonstrated that in *gpa6* mutant enlarged glutelin-containing DVs were mis-sorted to the apoplast, thus led to the reduction of PBII size.

### Map-based cloning of *GPA6*

**Fig. 5:**
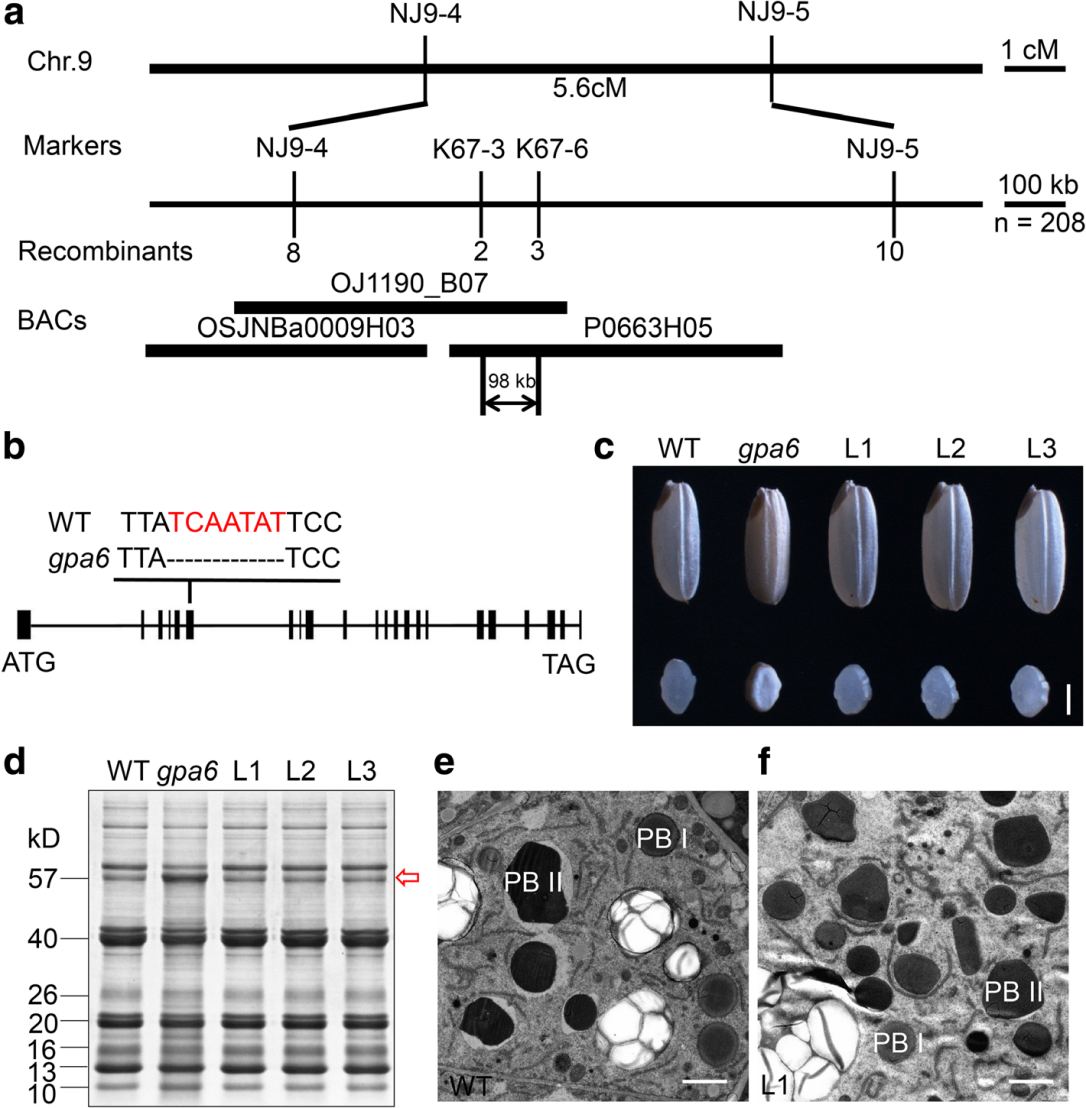
Map-based cloning of *GPA6*. **a** Fine mapping of the *GPA6* locus. The molecular markers and the number of recombinants are shown. **b** Gene structure and the mutation site in *Os09g0286400*. *Os09g0286400* comprises 22 exons (closed boxes) and 21 introns (lines). ATG and TGA represent the start and stop codons, respectively. A 7 bp deletion in the sixth exon of *Os09g0286400* in *gpa6*. **c**-**f** The *Os09g0286400* ORF under the control of *ubiquitin* promoter rescues the grain appearance (**c**), the storage protein composition pattern (**d**), the ultrastructures of endosperm cells in the wild type (**e**) and L1 (**f**). L1 to L3 denote the grains from three independent T1 transgenic lines. Red arrows in (**d**) indicate the 57-kD proglutelins. Bars = 1 mm in (**c**). Bars = 2 μm in (**e**) and (**f**)

The *gpa6* mutant was isolated from a ^60^Co-irradiated population of *indica* variety N22. Genetic analysis revealed that the mutant phenotype was inherited as a recessive mutation (Additional file 9: Table S2). For map-based cloning, we crossed *gpa6* with the *japonica* variety Nipponbare to generate 208 F_2_ recessive individuals. The *GPA6* locus was mapped to chromosome 9 and further fine-mapped to a 98-kb region (Fig. 5a). DNA sequencing revealed a 7 bp deletion in the sixth exon of *Os09g0286400*, generating a premature stop codon that led to a truncated product with 199 amino acids (Fig. 5b). Three independent transgenic lines bearing *Ubiquitin* promoter driven *Os09g0286400* open reading frame (ORF) rescued the *gpa6* mutant phenotypes, including the floury appearance of endosperm (Fig. 5c), the accumulation of proglutelins (Fig. 5d) and the abnormal glutelin deposit pattern (Fig. 5e and f). Therefore, *Os09g0286400* is the gene responsible for *gpa6* mutant phenotypes.

### *GPA6* encodes OsNHX5 that localizes to the Golgi, TGN and PVC

**Fig. 6:**
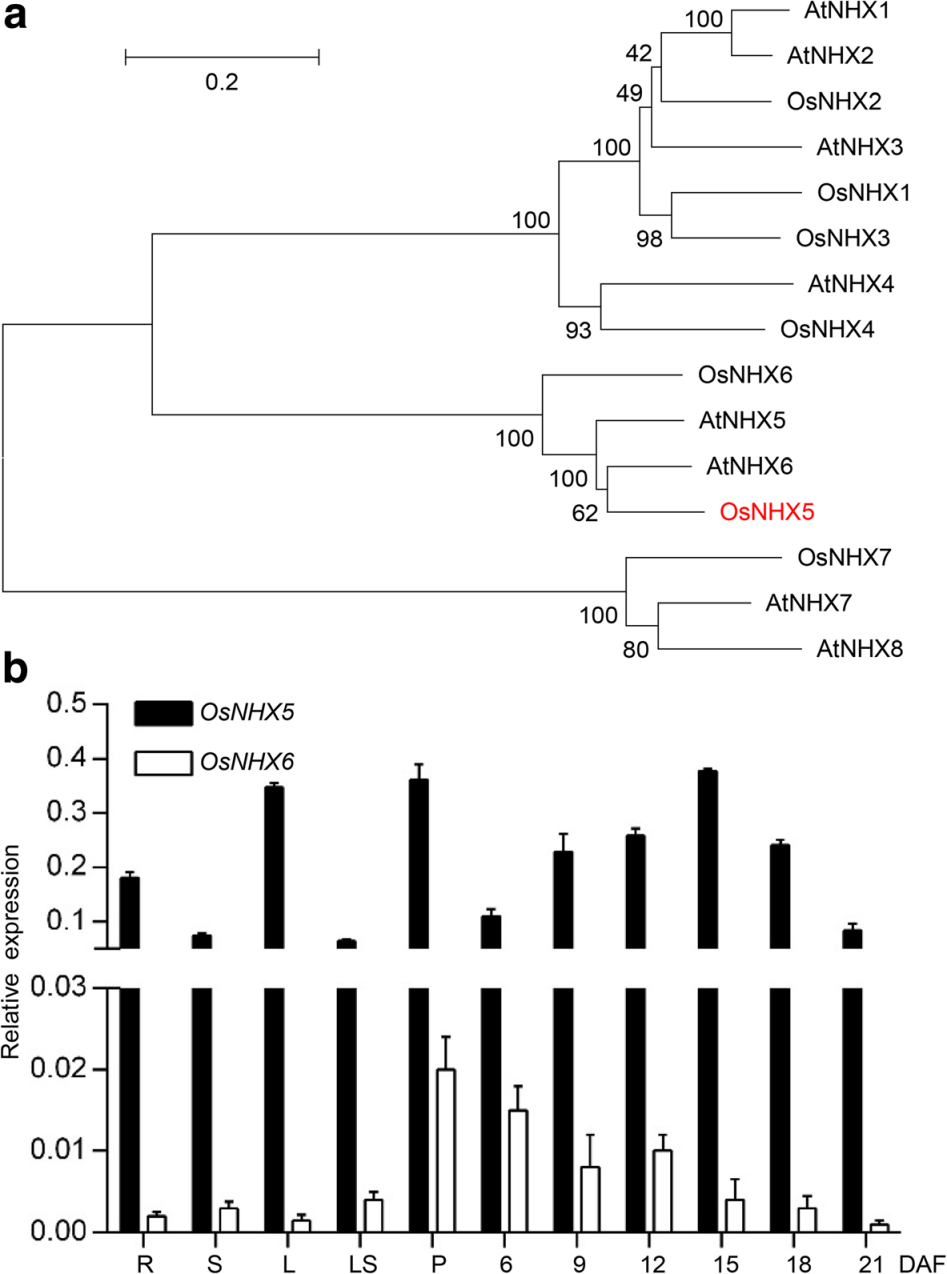
Phylogenetic analyses and spatial expression patterns of *OsNHX5*. **a** A neighbor-joining tree of OsNHX5 and its homologs. The tree was constructed using MEGA and bootstrapped with 1000 replicates. **b** Wild-type expression levels of *OsNHX5* and *OsNHX6* in various organs and different developmental stages of endosperm. R, root; S, stem; L, leaf; LS, leaf sheath; P, panicle; DAF, days after flowering. *Actin1* was used as an internal control. For each RNA sample, three technical replicates were performed. Values are means ± SD

*GPA6* encodes a NHX antiporter homologous to the endosomal localized *AtNHX5*, and was named *OsNHX5*. OsNHX5 is predicted to have 9 putative transmembrane domains and the mutation in *gpa6* leads to the deletion of the last four. Real-time PCR analysis revealed that *OsNHX5* is expressed in all tissues examined (Fig. 6b). During endosperm development, the expression of *OsNHX5* was low at the early stages, peaked at ~ 15-DAF, and decreased at ~ 18-DAF (Fig. 6b), which is correlated with the accumulation of glutelins. The rice genome has another putative endosomal antiporter OsNHX6 (Fig. 6a, Additional file 5: Figure S5). Although it is ubiquitiously expressed, its expression is much lower than *OsNHX5* (ratio of expression level of *OsNHX5*/*OsNHX6*, root: 91; stem: 25; leaf: 232; leaf sheath: 15; panicle: 18; 12-DAF endosperm: 26) (Fig. 6b).

**Fig. 7:**
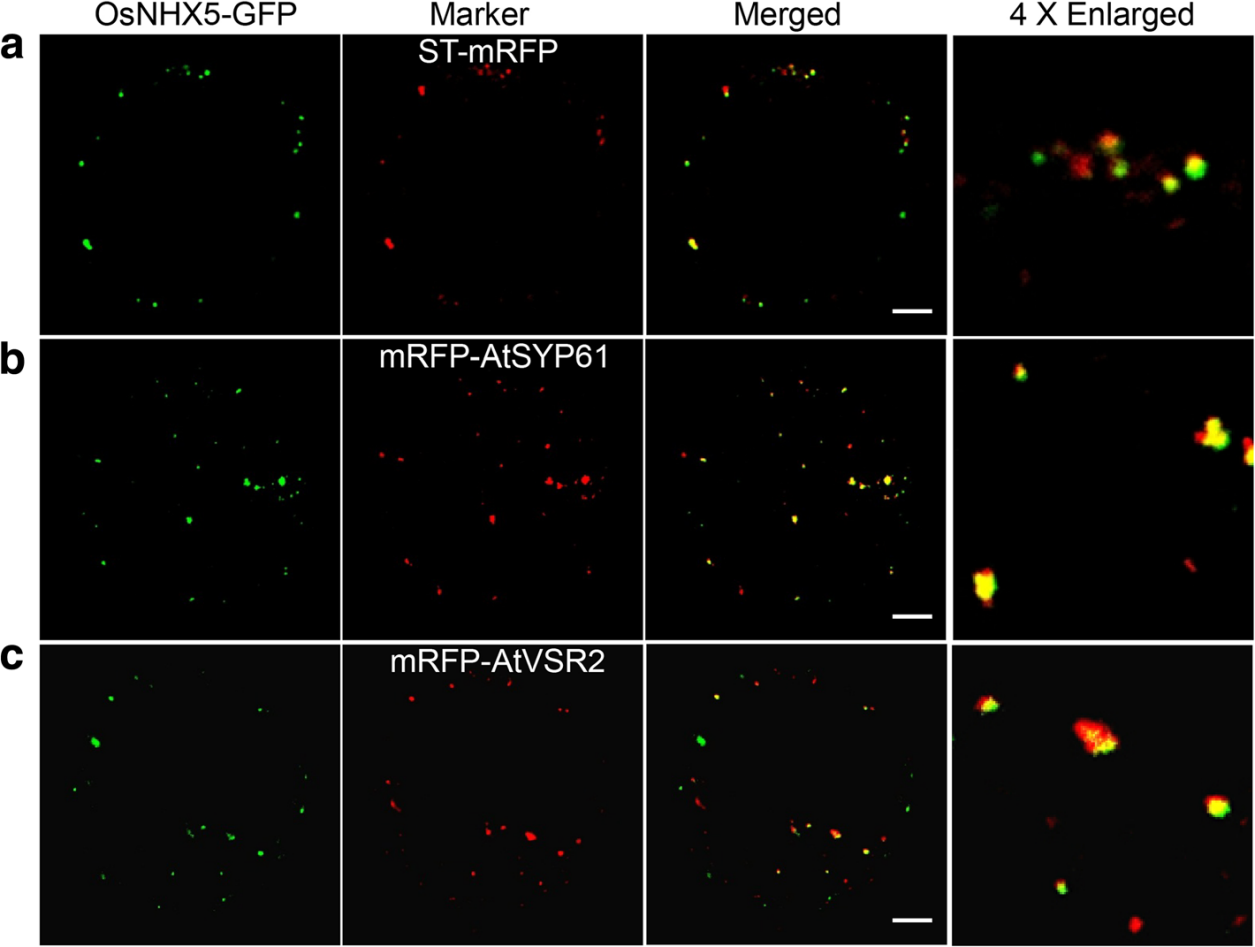
Subcellular Localization of OsNHX5 in *N. benthamiana* protoplasts. **a** to **c** Confocal microscopy images showing that OsNHX5-GFP is localized as punctate signals in the cytosol and its distribution partially overlaps with the markers for Golgi (ST-mRFP [**a**]), TGN (mRFP-SYP61 [**b**]) and PVC (mRFP-VSR2 [**c**]). Bars = 10 μm (**a**-**c**)

To determine the subcellular localization of OsNHX5, *OsNHX5* coding sequence was fused to the N-terminus of GFP to obtain *p35S:OsNHX5-GFP* construct. After transformed into *gpa6*, it completely rescued the mutant phenotype, indicating that OsNHX5-GFP is functional in vivo (Additional file 6: Figure S6). Unfortunately, GFP fluorescence in the transgenic lines was too weak to be detected. Transiently expressed *OsNHX5-GFP* in *N. benthamiana* leaf epidermal cells was examined instead, and OsNHX5-GFP was shown to partially colocalized with the Golgi, TGN and PVC markers, respectively (Pearson’s correlation coefficient [PSC], Golgi: PSC = 0.608 ± 0.08; TGN: PSC = 0.753 ± 0.14; PVC: PSC = 0.587 ± 0.11, Fig. 7). Similarly, OsNHX6 was localized to the Golgi, TGN, and PVC as well (Additional file 7: Figure S7).

Given the fact that *OsNHX5* mutation alone displayed phenotypes and *OsNHX6* is much lower expressed, OsNHX5 might be a predominant endosomal localized NHX antiporter in rice.

### The luminal pH of Golgi, TGN and PVC is more acidic in the *gpa6* protoplasts

**Fig. 8:**
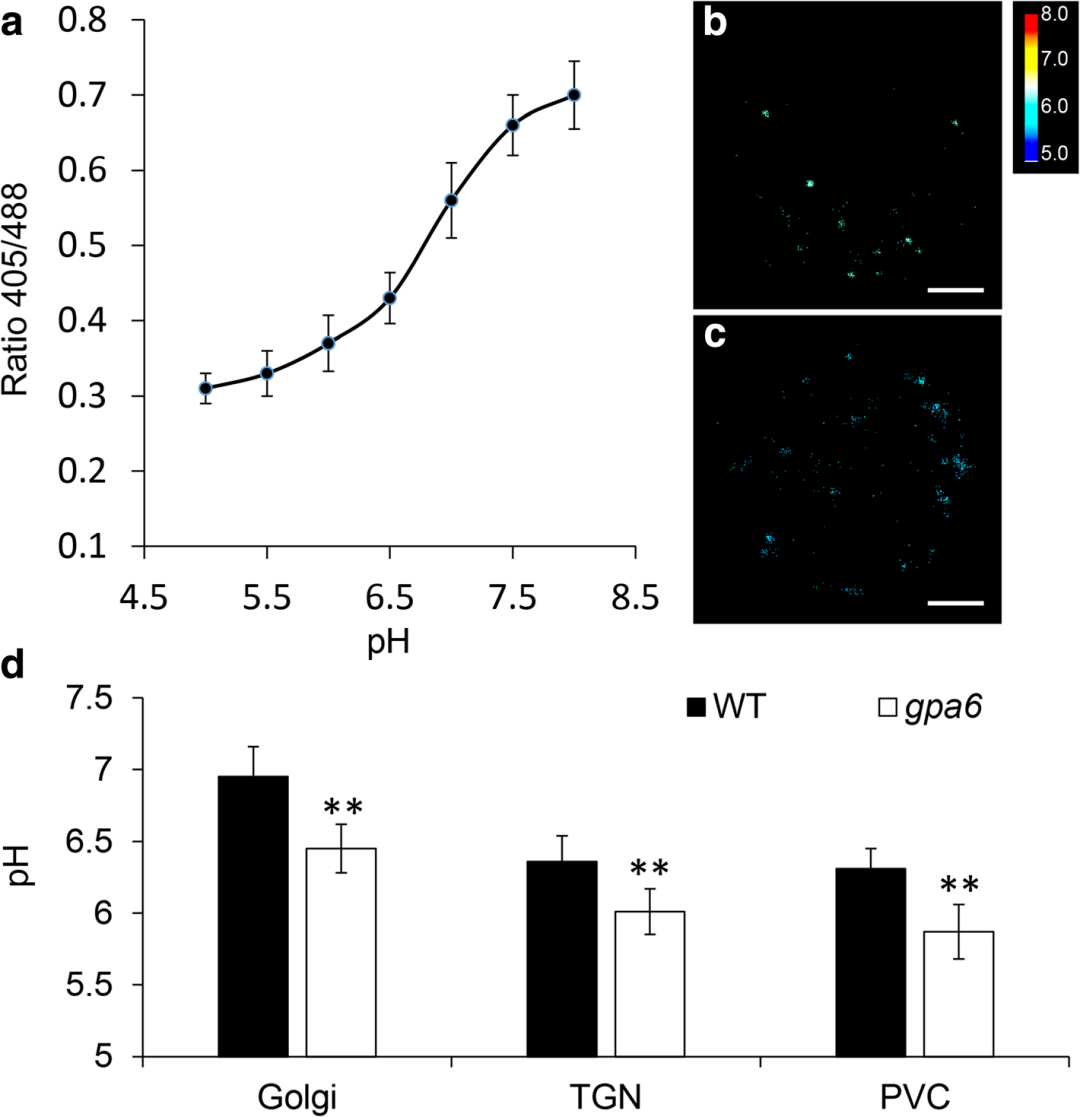
OsNHX5 regulates the pH of Golgi, TGN and PVC. **a** In vivo calibration curve of pH. pH calibration was achieved by equilibrating intracellular pH with 10 μM nigericin, 60 mM KCl, and 10 mM MES/HEPES Bis-Tris-propane, pH 5.0 to 8.0. (mean ± SD; *n* ≥ 25 protoplasts). **b** and **c** Representative pseudocolored images of PRpHluorin-BP80 (Y612A) in wild-type (**b**) or *gpa6* (**c**) protoplasts. Bars = 5 μm. **d** pH of the Golgi, TGN and PVC. (mean ± SD; *n* ≥ 25 protoplasts; **P < 0.01; Student’s *t* test)

In order to determine whether the cellular pH was affected in *gpa6*, we use noninvasive live-cell imaging to measure pH by the pHluorin-based pH sensor [37, 38]. pH sensors ManI-PRpHluorin, PRpHluorin-BP80 (Y612A) and PRpHluorin-AtVSR2 were used to measure pH of Golgi, TGN and PVC, respectively in the rice protoplasts [38]. The calibration curve was acquired by calculating pH-dependent fluorescence ratios (Fig. 8a). Our results showed that the pH of the Golgi, TGN, and PVC was more acidic in *gpa6* (Golgi: 6.45 ± 0.17; TGN: 6.01 ± 0.16; PVC: 5.87 ± 0.19) than in the wild type (Golgi: 6.95 ± 0.21; TGN: 6.36 ± 0.18; PVC: 6.31 ± 0.14) (Fig. 8d) where the △pH = 0.35–0.50. Representative pseudocoloured images of PRpHluorin-BP80 (Y612A) were shown (Fig. 8b and c). These results clearly indicated that OsNHX5 regulates the pH homeostasis of the Golgi, TGN, and PVC in rice protoplasts.

## Discussion

### *gpa6* is defective in post-Golgi trafficking of storage proteins in rice endosperm cells

Characterization of 57H mutants facilitates us to understand the molecular mechanism of proglutelin trafficking and processing in rice endosperm cells. In this study, we isolated a 57H mutant *gpa6* that accumulated large amount of proglutelins in rice endosperm. The comparable protein levels of BiP1 and PDI1–1 between wild type and the *gpa6* mutant suggested a normal ER function in *gpa6*. Consistent with this notion, *gpa6* developed normal ER-derived PBIs. In *gpa6* mutant, large amount of DVs accumulated in the cytosol, suggesting that the trafficking rate is largely reduced. Furthermore, DVs were mis-sorted to the apoplast, leading to smaller PBIIs. These abnormalities are very similar to *gpa1*/*osrab5a*, *gpa2*/*osvps9a*, and *gpa3*/*kelch* mutants [5, 6, 16, 18, 19]. Taken together, *gpa6* is defective in proglutelin post-Golgi vacuolar trafficking pathway in rice endosperm cells.

### OsNHX5 is a predominant intracellular NHX antiporter that regulates luminal pH of several endomembrane compartments

Similar to animals, plant cells also have specific pH in different endomembrane compartments along the secretory pathway [37–40]. In this study, using noninvasive live-cell imaging and pH sensors, we found that the pH values of Golgi, TGN and PVC in wild-type rice protoplasts were not significantly different from their counterparts in Arabidopsis protoplasts (Golgi: 6.8; TGN: 6.3; PVC: 6.2) [38]. However, all three compartments had a more acidic pH (△pH = 0.35–0.50) in *osnhx5*, indicating that OsNHX5 plays an important role in the alkalization of these compartments. Given the fact that *atnhx5 atnhx6* had a more acidic pH in those compartments as well (△pH = 0.25–0.40), endosomal antiporters seem to have conserved functions in rice and Arabidopsis cells.

In *atnhx5 atnhx6* double mutant, lowered pH led to a compromised receptor–cargo association [33]. The phenotype of storage protein trafficking defects were similar between *osnhx5* and *atnhx5 atnhx6*. Therefore, the acidification of Golgi, TGN, particularly PVC may result in reduced VSR (Vacuolar sorting receptor)-proglutelin association, although the receptors for proglutelins remains to be characterized. Previous studies showed that OsNHX5 has a K^+^ and Na^+^ transport activity [41], thus it is possible that ionic changes might also affect VSR-cargo interactions.

Previous studies implicate that maintaining pH homeostasis of TGN is required for protein trafficking in Arabidopsis [42]. DVs are unique carriers for proglutelin transport in rice presumably budded from the TGN [5, 6]. It is worth noting that the average size of DVs newly budded from TGN is enlarged to about 193 nm in *gpa6*, which is much bigger than 154 nm in the wild type (Fig. 3), 160 nm in *gpa2* [5], and 153 nm in *gpa3* mutants [6]. Thus, pH of TGN seems to have an impact on DV size control, although the detail mechanism remains to be explored.

## Conclusions

In summary, our studies demonstrated that OsNHX5 is localized to the Golgi, TGN and PVC to maintain pH homeostasis, which is important for DV-mediated glutelin trafficking in rice endosperm.

## Methods

### Plant materials and growth conditions

The *gpa6* mutant was identified from a ^60^Co-irradiated mutant pool of the *indica* cultivar N22 (Nagina22, an Indian traditional variety). An F_2_ population was produced from *gpa6* and a *japonica* variety Nipponbare for mapping. The seeds of all accessions were collected, stored and supplied by the State Key Laboratory of Crop Genetics and Germplasm Enhancement of Nanjing Agricultural University, Jiangsu, China. All plants were grown in the paddy field during the normal growing seasons or in a greenhouse at Nanjing, China. Developing seeds of wild type (N22) and *gpa6* at 6–21 days after fertilization (DAF) were used in the experiments.

### Protein Extraction from Rice seeds and immunoblot analysis

Total protein extraction and immunoblot assay were performed as described previously [16].

### Microscopy

Scanning electron microscopy, transmission electron microscopy, light and immunofluorescence microscopy and immunogold labeling analysis were performed as described previously [5, 6, 16, 17].

### Map-based cloning

To map the *GPA6* locus, an F_2_ population was generated from a cross between the *gpa6* mutant and a *japonica* variety Nipponbare. Total proteins were extracted from half of an individual rice seed and resolved by SDS-PAGE gel to monitor the accumulation of the proglutelins. Meanwhile, the other half of the identified mutant seeds with embryos was grown for DNA extraction. In total, 208 recessive individuals were used for fine mapping of *GPA6*. The primers used in fine mapping are listed in Additional file 10: Table S3.

### Real-time RT-PCR analysis

Total RNA was extracted from different tissues using an RNA Prep Pure Plant Kit (TIANGEN). First strand cDNA was synthesized using oligo (dT)18 as the primer (TaKaRa). Three biological replicates of real-time RT-PCR were performed with SYBR Premix Ex Taq II (TaKaRa) on an Applied Biosystems 7500 Real-Time PCR System. The primer sequences used for PCR are listed in Additional file 12: Table S5.

### Subcellular localization

For transient expression analysis in *N. benthamiana* leaf epidermal cells, the coding region of *OsNHX5* or *OsNHX6* was amplified and inserted into the binary vector pCAMBIA1305GFP to produce the *OsNHX5-GFP* or *OsNHX6-GFP* fusion construct (Additional file 11: Table S4). Construct were introduced into the Agrobacterium strain EHA105 and then used to infiltrate *N. benthamiana* leaves, as described previously [43]. *N. benthamiana* protoplasts were isolated using the same method used with Arabidopsis [44]. Fluorescence was observed using a confocal laser scanning microscope (Leica TCS-SP8).

### pH measurements

The rice protoplasts were isolated from 10-days-old N22 and *gpa6* seedlings. pH sensors ManI–PRpHluorin, PRpHluorin-BP80 (Y612A) and PRpHluorin–AtVSR2 were transformed into rice protoplasts as previously described [45, 46]. The PRpHluorin signals at emission wavelength of 500 to 550 nm were recorded with dual-excitation wavelength at 405 and 488 nm, respectively, and used to calculate the pH using the calibration curve. In vivo calibration was achieved from the same protoplasts expressing the PRpHluorin for pH measurement. Protoplasts were incubated in WI protoplast buffer (0.5 M mannitol and 20 mM KCl) with 25 μM nigericin, 60 mM KCl, and 10 mM MES/HEPES Bis-Tris-propane adjusted to different pH values ranging from 5.0 to 8.0 for each calibration point [33, 37, 38, 47–49]. Fluorescence was observed using a confocal laser scanning microscope (Leica TCS-SP8).

## Additional files

Additional file 1: **Figure S1.** Time-course analysis of storage proteins during endosperm development of the wild-type N22 and the mutant *gpa6*. (a) SDS-PAGE analyses of seed storage proteins during wild-type and gpa6 endosperm development. DAF, days after flowering. (b) Immunoblot analysis of glutelins during wild-type and gpa6 endosperm development. EF-1α was used as a loading control. Red arrows in (a) and (b) indicate the 57-kD proglutelins. (DOCX 235 kb)

Additional file 2: **Figure S2.** RT-qPCR assay of the expression of representative genes coding for storage proteins in 12-DAF endosperm. Glutelin genes: GluA1, GluB2, GluC1, GluD1; prolamin genes: pro10.1, pro16.2, pro13a.2, pro13b.2. Values are means ± SD. *n* = 3. (DOCX 81 kb)

Additional file 3: **Figure S3.** Immunofluorescence microscopy of protein bodies in the subaleurone cells of the wild type and *gpa6* mutant. (a) to (f) Immunofluorescence microscopy images of storage proteins in wild-type (a-c) and *gpa6* (d-f) 12 DAF seeds. (a, d) Secondary antibodies conjugated with Alexa fluor 555 (red) were used to trace the antigens recognized by the anti-α-globulin antibodies. (b, e) Secondary antibodies conjugated with Alexa fluor 488 (green) were used to trace the antigens recognized by the anti-glutelin antibodies. (c, f) Merged images. White arrowheads in (f) indicate the mis-sorted α-globulins in the PMB. Bars = 10 μm (a-f). (DOCX 135 kb)

Additional file 4 **Figure S4.** Distribution of cell wall materials in 12 DAF endosperm cells. (a) to (f) Sections of 12 DAF endosperms from wild type (a-c) and gpa6 (d-f) plant were incubated with pectin (JIM7) or glutelin antibodies, followed by secondary antibodies conjugated to Alexa-555 or Alexa-488. Bars = 10 μm (a-f). (DOCX 152 kb)

Additional file 5: **Figure S5.** Amino acid sequences alignment of OsNHX5 and OsNHX6. (DOCX 421 kb)

Additional file 6: **Figure S6.** Complementation of *gpa6* mutant phenotypes by *p35S:OsNHX5-GFP*. (a) Immunoblot analysis with monoclonal GFP antibodies. (b) p35S:OsNHX5-GFP transgene rescued the grain phenotype of the gpa6 mutant. Bars = 1 mm. (c) p35S:OsNHX5-GFP transgene in the gpa6 mutant reduce the amount of 57-KD proglutelins to a level comparable to the wild type. GL1 to GL3 denote the grains from three independent T1 transgenic lines. Red arrows indicate the 57-kD proglutelins. (DOCX 186 kb)

Additional file 7: **Figure S7.** Subcellular Localization of OsNHX6 in *N. benthamiana* protoplasts. (a) to (c) Confocal microscopy images showing that OsNHX6-GFP is localized as punctate signals in the cytosol and its distribution partially overlaps with the markers for Golgi (ST-mRFP [a]), TGN (mRFP-SYP61 [b]) and PVC (mRFP-VSR2 [c]). Bars = 10 μm (a-c). (DOCX 112 kb)

Additional file 8: **Table S1.** Properties of wild-type and *gpa6* seeds. (DOCX 13 kb)

Additional file 9: **Table S2.** Segregation of mutant phenotypes in reciprocal crosses between the wild type and *gpa6* mutant. (DOCX 13 kb)

Additional file 10: **Table S3.** Primers used for mapping. (DOCX 14 kb)

Additional file 11: **Table S4.** Primers used for vector construction. (DOCX 14 kb)

Additional file 12: **Table S5.** Primer used for real-time PCR analysis. (DOCX 14 kb)

## Data Availability

All data generated or analyzed during this study are included in this published article and its additional files.

## Abbreviations

DV: Dense vesicle
ER: Endoplasmic reticulum
GPA: Glutelin precursor accumulation
PBI: Protein body I
PBII: Protein body II
PMB: Paramural body
PSV: Protein storage vacuole
PVC: Prevacuolar compartment
TGN: *Trans*-Golgi network
