# Supplementary material for: OsNHX5-mediated pH homeostasis is required for post-Golgi trafficking of seed storage proteins in rice endosperm cells
Source: BMC Plant Biol. 2019 Jul 5;19:295. doi: 10.1186/s12870-019-1911-y (PMC6612104; doi:10.1186/s12870-019-1911-y)
Supplement: Supplementary file 1 — Figure S1. Time-course analysis of storage proteins during endosperm development of the wild-type N22 and the mutant gpa6. (a) SDS-PAGE analyses of seed storage proteins during wild-type and gpa6 endosperm development. DAF, days after flowering. (b) Immunoblot analysis of glutelins during wild-type and gpa6 endosperm development. EF-1α was used as a loading control. Red arrows in (a) and (b) indicate the 57-kD proglutelins. (DOCX 235 kb) [file 12870_2019_1911_MOESM1_ESM.docx]

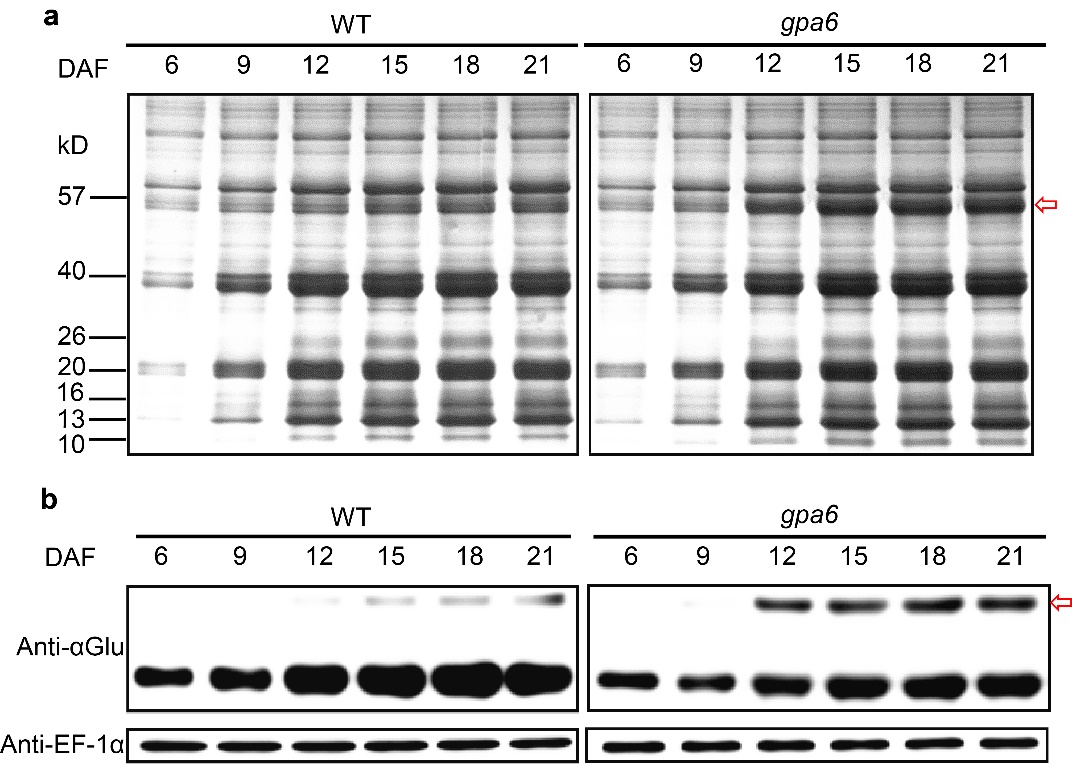


**Figure S1.** Time-course analysis of storage proteins during endosperm development of the wild-type N22 and the mutant *gpa6*.
