# Supplementary material for: OsNHX5-mediated pH homeostasis is required for post-Golgi trafficking of seed storage proteins in rice endosperm cells
Source: BMC Plant Biol. 2019 Jul 5;19:295. doi: 10.1186/s12870-019-1911-y (PMC6612104; doi:10.1186/s12870-019-1911-y)
Supplement: Supplementary file 2 — Figure S2. RT-qPCR assay of the expression of representative genes coding for storage proteins in 12-DAF endosperm. Glutelin genes: GluA1, GluB2, GluC1, GluD1; prolamin genes: pro10.1, pro16.2, pro13a.2, pro13b.2. Values are means ± SD. n = 3. (DOCX 81 kb) [file 12870_2019_1911_MOESM2_ESM.docx]

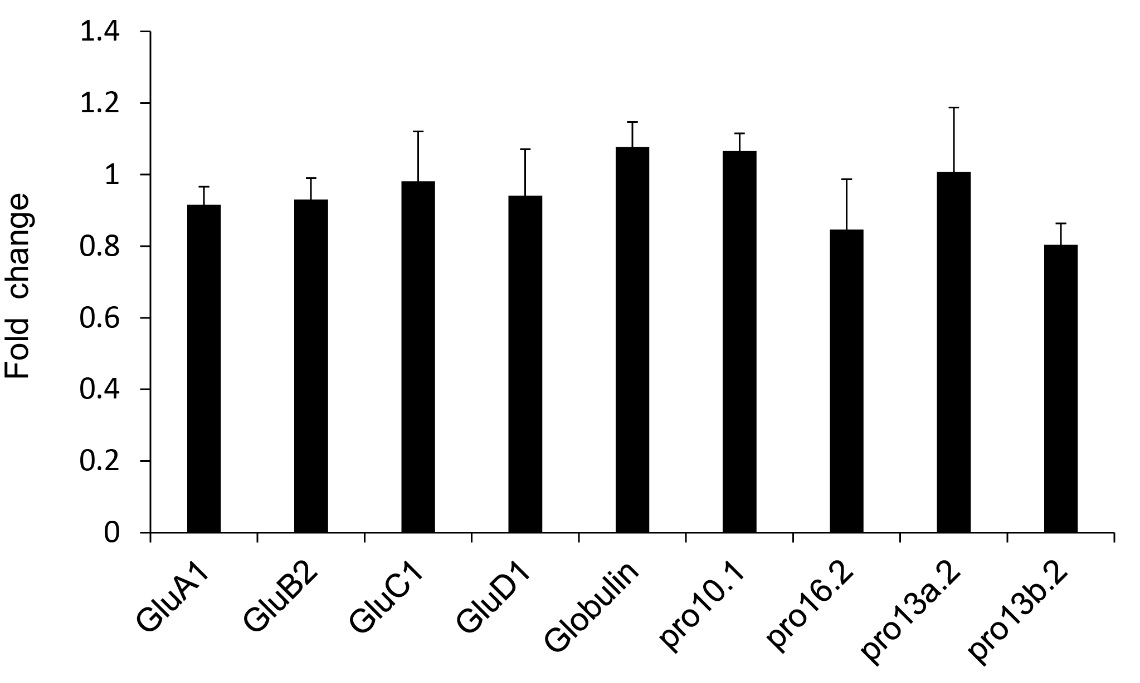


**Figure S2.** RT-qPCR assay of the expression of representative genes coding for storage proteins in 12-DAF endosperm.
