# Supplementary material for: OsNHX5-mediated pH homeostasis is required for post-Golgi trafficking of seed storage proteins in rice endosperm cells
Source: BMC Plant Biol. 2019 Jul 5;19:295. doi: 10.1186/s12870-019-1911-y (PMC6612104; doi:10.1186/s12870-019-1911-y)
Supplement: Supplementary file 3 — Figure S3. Immunofluorescence microscopy of protein bodies in the subaleurone cells of the wild type and gpa6 mutant. (a) to (f) Immunofluorescence microscopy images of storage proteins in wild-type (a-c) and gpa6 (d-f) 12 DAF seeds. (a, d) Secondary antibodies conjugated with Alexa fluor 555 (red) were used to trace the antigens recognized by the anti-α-globulin antibodies. (b, e) Secondary antibodies conjugated with Alexa fluor 488 (green) were used to trace the antigens recognized by the anti-glutelin antibodies. (c, f) Merged images. White arrowheads in (f) indicate the mis-sorted α-globulins in the PMB. Bars = 10 μm (a-f). (DOCX 135 kb) [file 12870_2019_1911_MOESM3_ESM.docx]

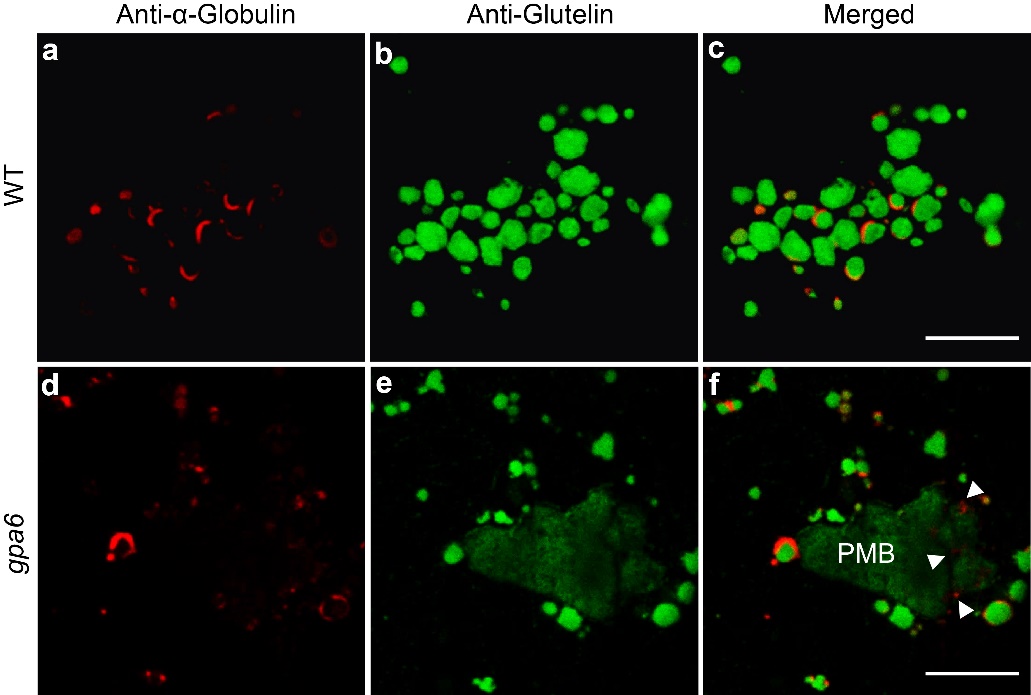


**Figure S3.** Immunofluorescence microscopy of protein bodies in the subaleurone cells of the wild type and *gpa6* mutant.

(a) to (f) Immunofluorescence microscopy images of storage proteins in wild-type (a-c) and *gpa6* (d-f) 12 DAF seeds. (a, d) Secondary antibodies conjugated with Alexa fluor 555 (red) were used to trace the antigens recognized by the anti-α-globulin antibodies. (b, e) Secondary antibodies conjugated with Alexa fluor 488 (green) were used to trace the antigens recognized by the anti-glutelin antibodies. (c, f) Merged images. White arrowheads in (f) indicate the mis-sorted α-globulins in the PMB. Bars = 10 μm (a-f).
