# Supplementary figures and images for: OsNHX5-mediated pH homeostasis is required for post-Golgi trafficking of seed storage proteins in rice endosperm cells
Source: BMC Plant Biol. 2019 Jul 5;19:295. doi: 10.1186/s12870-019-1911-y (PMC6612104; doi:10.1186/s12870-019-1911-y)

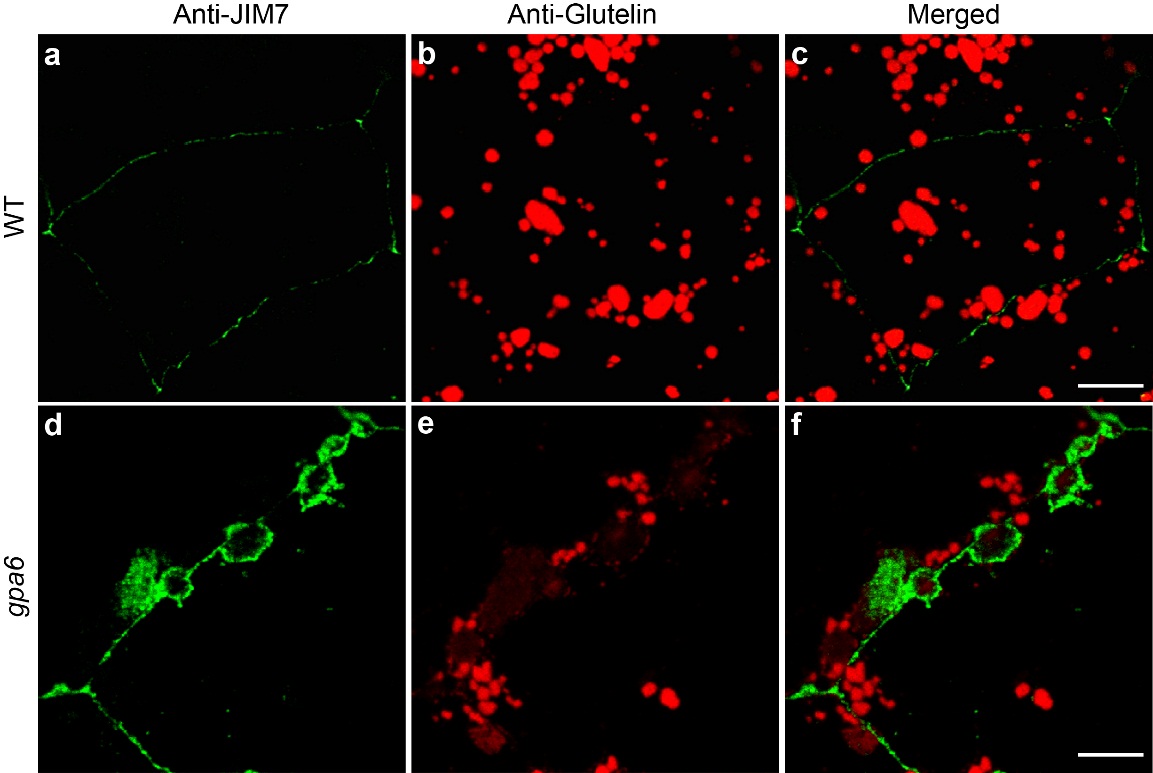


**Figure S4.** Distribution of cell wall materials in 12 DAF endosperm cells.

Supplement: Supplementary file 4 — Figure S4. Distribution of cell wall materials in 12 DAF endosperm cells. (a) to (f) Sections of 12 DAF endosperms from wild type (a-c) and gpa6 (d-f) plant were incubated with pectin (JIM7) or glutelin antibodies, followed by secondary antibodies conjugated to Alexa-555 or Alexa-488. Bars = 10 μm (a-f). (DOCX 152 kb) [file 12870_2019_1911_MOESM4_ESM.docx]

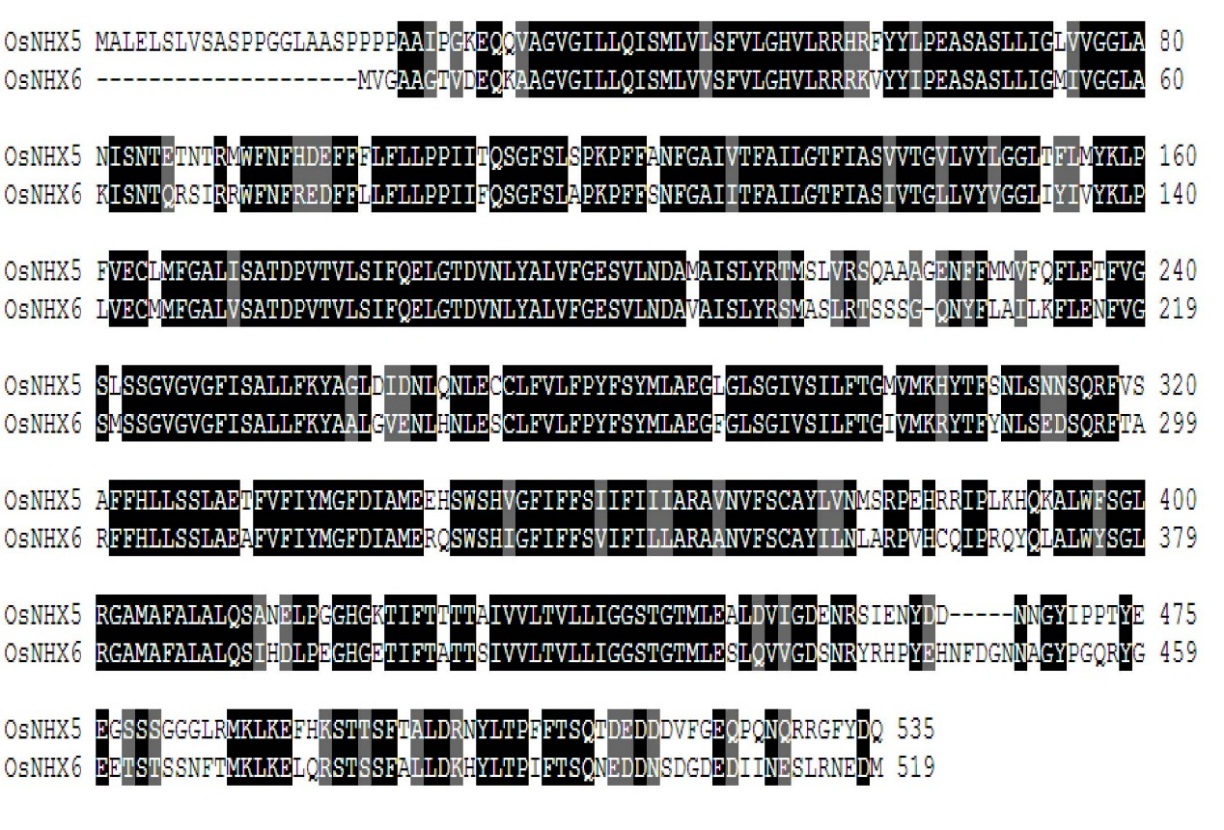


**Figure S5.** Amino acid sequences alignment of OsNHX5 and OsNHX6.

Supplement: Supplementary file 5 — Figure S5. Amino acid sequences alignment of OsNHX5 and OsNHX6. (DOCX 421 kb) [file 12870_2019_1911_MOESM5_ESM.docx]

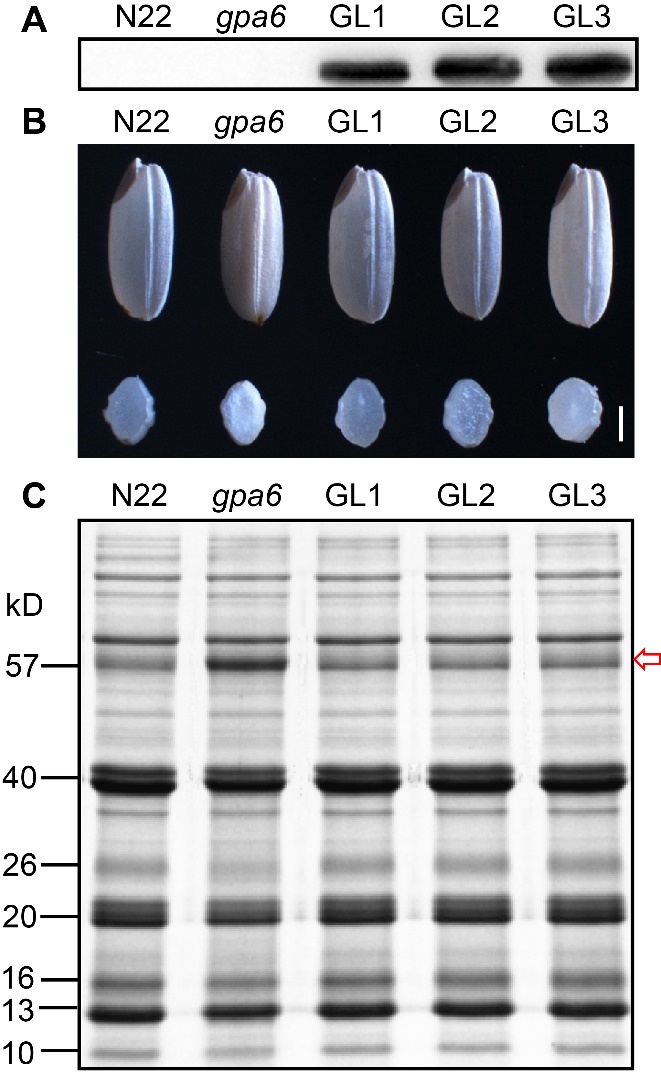


**Figure S6.** Complementation of *gpa6* mutant phenotypes by *p35S:OsNHX5-GFP*.

Supplement: Supplementary file 6 — Figure S6. Complementation of gpa6 mutant phenotypes by p35S:OsNHX5-GFP. (a) Immunoblot analysis with monoclonal GFP antibodies. (b) p35S:OsNHX5-GFP transgene rescued the grain phenotype of the gpa6 mutant. Bars = 1 mm. (c) p35S:OsNHX5-GFP transgene in the gpa6 mutant reduce the amount of 57-KD proglutelins to a level comparable to the wild type. GL1 to GL3 denote the grains from three independent T1 transgenic lines. Red arrows indicate the 57-kD proglutelins. (DOCX 186 kb) [file 12870_2019_1911_MOESM6_ESM.docx]

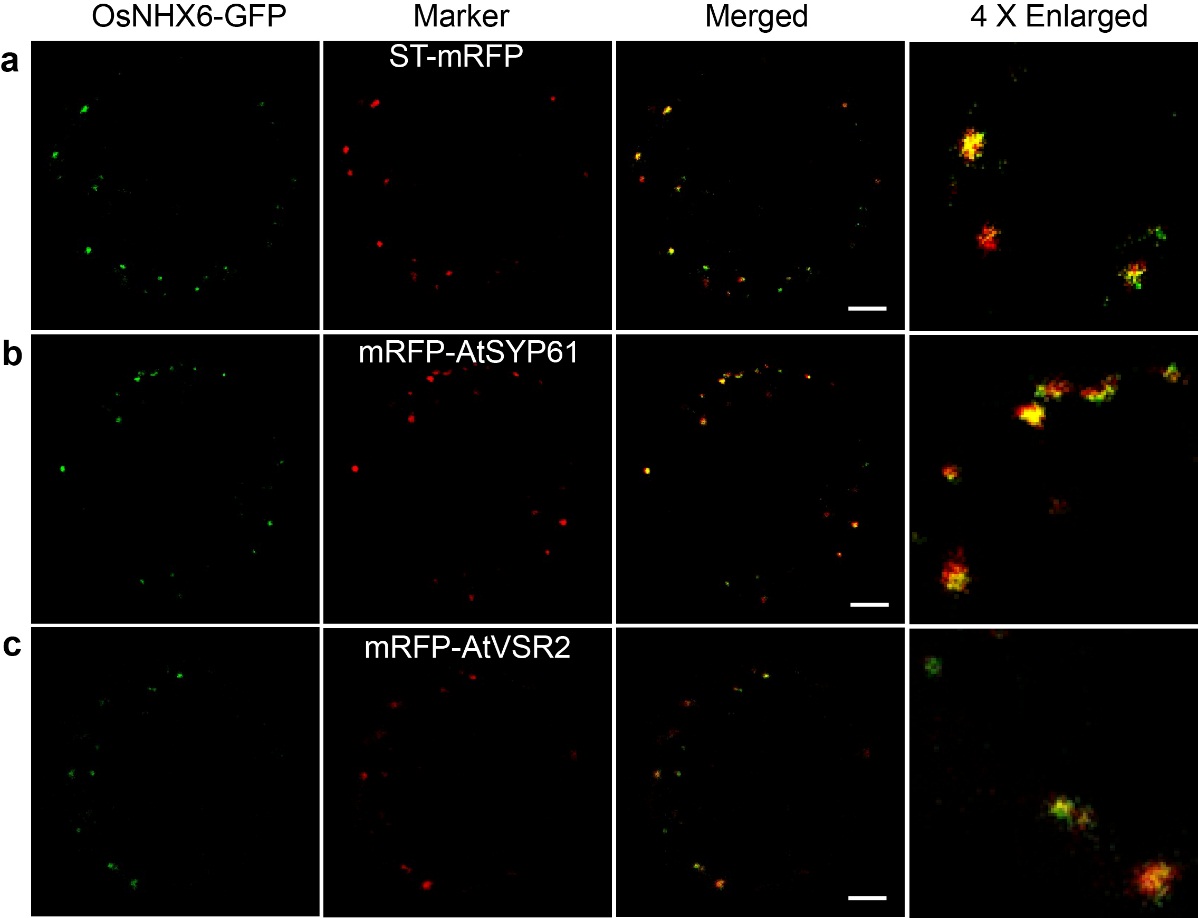


**Figure S7.** Subcellular Localization of OsNHX6 in *N. benthamiana* protoplasts.

Supplement: Supplementary file 7 — Figure S7. Subcellular Localization of OsNHX6 in N. benthamiana protoplasts. (a) to (c) Confocal microscopy images showing that OsNHX6-GFP is localized as punctate signals in the cytosol and its distribution partially overlaps with the markers for Golgi (ST-mRFP [a]), TGN (mRFP-SYP61 [b]) and PVC (mRFP-VSR2 [c]). Bars = 10 μm (a-c). (DOCX 112 kb) [file 12870_2019_1911_MOESM7_ESM.docx]
