# Supplementary material for: OsNHX5-mediated pH homeostasis is required for post-Golgi trafficking of seed storage proteins in rice endosperm cells
Source: BMC Plant Biol. 2019 Jul 5;19:295. doi: 10.1186/s12870-019-1911-y (PMC6612104; doi:10.1186/s12870-019-1911-y)
Supplement: Supplementary file 8 — Table S1. Properties of wild-type and gpa6 seeds. (DOCX 13 kb) [file 12870_2019_1911_MOESM8_ESM.docx]

**Table S1.** **Properties of wild-type and *gpa6* seeds.**

|  | WT | *gpa6* |
| --- | --- | --- |
| 1,000-grain weight (g) | 19.08 ± 0.15 | 14.68 ± 0.11** |
| Protein content (%) | 11.31 ± 0.06 | 11.23 ± 0.04 |
| Amylose content (%) | 25.15 ± 0.17 | 20.02 ± 0.13** |
| Lipid content (%) | 2.05 ± 0.06 | 3.72 ± 0.07** |

Values are means ± SD. ***P* < 0.01 (Student’s *t* test).
