# Supplementary material for: OsNHX5-mediated pH homeostasis is required for post-Golgi trafficking of seed storage proteins in rice endosperm cells
Source: BMC Plant Biol. 2019 Jul 5;19:295. doi: 10.1186/s12870-019-1911-y (PMC6612104; doi:10.1186/s12870-019-1911-y)
Supplement: Supplementary file 9 — Table S2. Segregation of mutant phenotypes in reciprocal crosses between the wild type and gpa6 mutant. (DOCX 13 kb) [file 12870_2019_1911_MOESM9_ESM.docx]

**Table S2.** **Segregation of mutant phenotypes in reciprocal crosses between the wild type and *gpa6* mutant.**

| Cross | Normal | 57H/Floury | X^2^_3:1_ |
| --- | --- | --- | --- |
| *gpa6*/wild type F_2_ | 165 | 51 | 0.15 |
| wild type/*gpa6* F_2_ | 145 | 44 | 0.21 |
