# Supplementary material for: OsNHX5-mediated pH homeostasis is required for post-Golgi trafficking of seed storage proteins in rice endosperm cells
Source: BMC Plant Biol. 2019 Jul 5;19:295. doi: 10.1186/s12870-019-1911-y (PMC6612104; doi:10.1186/s12870-019-1911-y)
Supplement: Supplementary file 10 — Table S3. Primers used for mapping. (DOCX 14 kb) [file 12870_2019_1911_MOESM10_ESM.docx]

**Table S3.** **Primers used for mapping.**

| Primer name | Forward  sequence | Reverse  sequence |
| --- | --- | --- |
| N9-14 | CGACGAACTCCTCTACCGTTTACC | CTGCGTGTATCCAATCCCAAGG |
| NJ9-21 | CTCTCTGACGATGACCCTCTC | GGATGCTTCTGGGGCTATG |
| NJ9-4 | GCATACTGCTTTTACGACAA | CTGCCACTGGGATTAGAA |
| NJ9-5 | GAATCGTATTGCCAGCG | AAATCAGAGAAGTAGAGAGCGT |
| NJ9-14 | TGCTTCTGTTGCTGCC | CGAAACTCTTGTAGTCGTCA |
| K67-3 | TCATCATGCCTGCAATGCC | GGCGCCACAAATCTAGCAAT |
| K67-6 | AGGATTTAAGTCACTATGCTCCTT | CATATGTGTTGGGTGCGACA |
