# Supplementary material for: OsNHX5-mediated pH homeostasis is required for post-Golgi trafficking of seed storage proteins in rice endosperm cells
Source: BMC Plant Biol. 2019 Jul 5;19:295. doi: 10.1186/s12870-019-1911-y (PMC6612104; doi:10.1186/s12870-019-1911-y)
Supplement: Supplementary file 11 — Table S4. Primers used for vector construction. (DOCX 14 kb) [file 12870_2019_1911_MOESM11_ESM.docx]

**Table S4.** **Primers used for vector construction.**

| Usage | Primer name | Sequence |
| --- | --- | --- |
| Ubi-  OsNHX5 | 1390-OsNHX5-F | CCGGCGCGCCAAGCTTATGGCGCTGGAGCTGAGCCTG |
|  | 1390-OsNHX5-R | GAATTCCCGGGGATCCCTATTGGTCATAGAATCCGCG |
| Subcellular  localization | 1305-OsNHX5-GFP-F | CGGAGCTAGCTCTAGAATGGCGCTGGAGCTGAGCCTG |
|  | 1305-OsNHX5-GFP-R | TGCTCACCATGGATCCTTGGTCATAGAATCCGCG |
| Subcellular | 1305-OsNHX6-GFP-F | CGGAGCTAGCTCTAGAATGGTGGGGGCGGCGGGG |
| localization | 1305-OsNHX6-GFP-R | TGCTCACCATGGATCCCATATCCTCATTCCTCAATG |
