# Supplementary material for: OsNHX5-mediated pH homeostasis is required for post-Golgi trafficking of seed storage proteins in rice endosperm cells
Source: BMC Plant Biol. 2019 Jul 5;19:295. doi: 10.1186/s12870-019-1911-y (PMC6612104; doi:10.1186/s12870-019-1911-y)
Supplement: Supplementary file 12 — Table S5. Primer used for real-time PCR analysis. (DOCX 14 kb) [file 12870_2019_1911_MOESM12_ESM.docx]

**Table S5.** **Primer used for real-time PCR analysis.**

| Primer name | Forward  sequence | Reverse  sequence |
| --- | --- | --- |
| GluA1 | TGATGGTGAAGTGCCAGTTGTTGC | ACGCCTGTATGCTTGAGGGTTTCT |
| GluB2 | TGTCCTTCGCCCTGGACAACTATT | TGGTAAGGCGCGGAATACTGAGTT |
| GluC1 | ATGTAAAGTTCAACCGCGGCGATG | TCTCGTTGATCTGCCACTCAGCAT |
| GluD1 | AAATGAGCAGTTTCGATGCGCTGG | TGTTCGAGTATCGAGGCACCACAA |
| Pro10.1 | GTTGCATGCAGCTACAAGGCATGA | TCTGCATCGCCATCTTCACCATCT |
| Pro13a.2 | TTCAGGCGATTGTGCAGCAACTAC | GGATGGCAAGTTTAAGGCCAGCAA |
| Pro13b.2 | GTATAGCATTGCGGCAAGCACCTT | ATGGCCTGGACAACGTTAATGTCC |
| Pro16.2 | GCTCTCAATTTGCCCTCCATGTGT | TGGTACACACTACCAAGAACCGCA |
| Globulin | GATCAGTTCATCACCAACAAAACA | TCCAGAACGCACAAAATCAT |
| OsNHX5 | TTTGCACTTGCTCTCCAATCTGC | TCGATCCTCCGATAAGAAGTACCG |
| OsNHX6 | ATGGTGGGGGCGGCGGGGACG | CGTGGCCGAGCACGAAGGAG |
| Actin1 | TGGTCGTACCACAGGTATTG | CCACATCTGCTGGAATGTGCTG |
